# Depot-specific differences in perilipin and hormone-sensitive lipase expression in lean and obese

**DOI:** 10.1186/1476-511X-8-58

**Published:** 2009-12-18

**Authors:** Hind Ray, Claudie Pinteur, Vincent Frering, Michel Beylot, Valérie Large

**Affiliations:** 1 Department of Pathology and Cell Biology, Institute for Research in Immunology and Cancer, Université de Montréal, PO Box 6128, Station Centre-ville, Montréal Québec, H3C 3J7, Canada; 2 Institut National de la Santé et de la Recherche Médicale, U820, Lyon, F-69008 France; 3 Clinique de la Sauvegarde, Lyon, France; 4 Institut National de la Santé et de la Recherche Médicale, ERI22 EA4173, Lyon, F-69008 France; 5 Institut National de la Santé et de la Recherche Médicale, U855, Lyon, F-69008 France; 6 Université de Lyon, Lyon, F-69008 France; 7 Université Lyon 1, Villeurbanne, F-69622 France; 8 Institut National de la Santé et de la Recherche Médicale, IFR 62 Lyon-Est, Lyon, F-69008 France

## Abstract

**Background:**

Mainly dependent on hormone-sensitive lipase, lipolysis is differently impaired between fat depots in human obesity. Perilipin A expression is a critical element in adipocyte lipolysis. The present study aimed at comparing expression and subcellular distribution of perilipin and hormone-sensitive lipase in two abdominal adipose tissues of lean and obese women. We examined whether regional differences in perilipin expression contribute to impaired lipolytic rates.

**Methods:**

Abdominal subcutaneous and omental adipose tissues were obtained from six lean and ten obese women. We measured total protein content and relative distribution of hormone-sensitive lipase and perilipin proteins between lipid and non-lipid fractions in tissue homogenates. Hormone-sensitive lipase and perilipin mRNA levels, adipocyte size, basal (non-stimulated) and noradrenaline-stimulated lipolysis in isolated adipocytes were determined.

**Results:**

Adipocytes were significantly larger in the obese versus the lean women and in subcutaneous versus omental fat. Expressed as a function of cell number, basal lipolysis and noradrenaline responsiveness were higher in subcutaneous versus omental adipocytes from the obese women (P < 0.05). Despite higher or identical mRNA levels in the lean and the obese subjects and in subcutaneous and omental tissues, perilipin protein expression was lower in both depots in the obese versus the lean women, and in subcutaneous versus omental in both lean and obese women (P < 0.05). Perilipin was mostly (above 80%) present in the lipid fraction in both depots from the obese patients and the value decreased to 60% in the lean subjects (P < 0.05). Perilipin protein expression was inversely correlated to adipocyte size and basal lipolysis in both depots. Despite higher mRNA levels, hormone-sensitive lipase protein expression decreased in both depots of the obese women. Regional difference for hormone-sensitive lipase was reported in lipid fraction of subcutaneous fat of the obese subjects: hormone-sensitive lipase content was twice as low as in omental adipose tissue.

**Conclusion:**

In both fat depots, a reduced perilipin protein expression was observed in women obesity. Perilipin protein level may contribute to differences in basal lipolysis and in adipocyte size between fat depots and may regulate lipid accumulation in adipocytes. Differences in hormone-sensitive lipase subcellular distribution were reported between fat depots in the obese women.

## Background

Obesity results from a long term imbalance between storage and mobilization of triacylglycerols (TAGs) in adipose tissue [1]. It is commonly accepted that the ability to stimulate lipolysis (TAG mobilization) is impaired in obese adipose tissue [2]. Resistance to catecholamines-induced lipolysis is demonstrated in abdominal subcutaneous (SC) fat of obese subjects [3]. A more distal defect in catecholamine signal transduction at the level of hormone-sensitive lipase (HSL, the rate-limiting enzyme for hydrolysis of stored TAGs) is observed in SC fat of obese women and is linked to decreased expression and activity of HSL [4].

It is now apparent that other specific proteins located at the surface of lipids droplets i.e. the perilipin family have also an important role in the control of lipolysis. The spliced isoforms of perilipins A (*PLIN*) and B are found in adipocytes, the A form being largely predominant in mature adipocytes [3]. In the basal state (in the absence of stimulation of lipolysis), perilipins are mainly present on the surface of lipid droplets and prevent LHS from TAGs hydrolysis [5]. When lipolysis is stimulated by β-adrenergic agents, perilipins phosphorylation in response to PKA activation induces an important physical alteration of the droplet surface that facilitates the action of phosphorylated lipases such as HSL and initiates lipolysis [6-8]. *PLIN *suppression in mice increases basal lipolysis (rate of lipolysis under unstimulated conditions) and prevents the development of obesity induced by a high fat diet [9]. *PLIN *ablation reverses obesity in db/db mice [10]. Evidence for a role of perilipin in humans is also provided [11-13]. These studies confirm the presence of perilipin A in human adipose tissue. Low perilipin content in abdominal SC adipose tissue is associated with a high basal lipolytic rate of isolated adipocytes [11,12].

Human adipose tissue displays significant regional differences in adipocyte size, basal metabolic activities and hormonal responsiveness [3]. These differences probably contribute to regional fat deposition. Regional differences in regulation of lipolysis were shown *in vitro *and *in vivo *[3,14]. The interpretation of these studies is complex and generates equivocal data since it depends on gender, fat depot (intra-abdominal and more peripheral depots), body mass index (BMI) and the choice of lipolysis units [3,15-18]. Abdominal adipocytes are generally believed to be hyperlipolytic by being highly responsive to catecholamine stimulation [17,19]. Most studies using isolated adipocytes show higher basal lipolysis in SC versus omental (OM) adipocytes [17,20,21]. Regarding HSL expression, a decrease is reported in SC depot of obese subjects versus lean subjects [4]. Despite the crucial role of the adipose tissue heterogeneity, few studies only attempt to compare expression of HSL and perilipin in different fat depots. The present report aimed at examining whether HSL and perilipin A were differently expressed in abdominal SC and OM adipose tissues of women and if these expressions and regional differences were modified in obese subjects.

## Methods

### Subjects

Written informed consent was obtained from 10 obese and six lean women. All of them had normal physical examination and none took any medication. The study was approved by the local ethical committee and the French National Institute of Health and Medical Research (INSERM). Samples of abdominal SC and OM adipose tissues were collected at the beginning of surgery in obese subjects and in three lean subjects under general anesthesia with propofol. The SC adipose tissue biopsies were obtained by needle aspiration under local anesthesia with lidocaïne in an additional group of three lean subjects. Previous data have shown that the adrenergic regulation of lipolysis is not influenced by the mode of sampling in SC fat cells. Therefore we decided to pool data generated from both forms of anesthesia [22]. All samples were collected in the post-absorptive state. They were frozen in liquid nitrogen and stored at -80°C until analysis, except for a small part of the biopsies that was used for measuring fat adipocyte volume.

### Preparation of isolated fat cells and determination of adipocyte volume

Isolated fat cells were prepared with collagenase according to Rodbell [15]. An aliquot of cells was suspended in an albumin buffer solution and placed on a glass slide to determine the diameter of 100 cells with a microscope (Carl Zeiss, Inc. Thornwood, NY) equipped with a caliper scale. Mean fat cell volume was calculated according to the methods developed by Hirsch and Gallian [23].

### Lipolysis experiments

Isolated fat cells from adipose tissue biopsies were incubated as described in detail [17]. In brief about 5-10,000 cells per ml isolated by collagenase treatment, were incubated in Krebs-Ringer phosphate (pH 7.4) containing albumin (20 g/L); glucose (1 g/L) and ascorbic acid (0.1 g/L) in the absence or in the presence of increasing concentrations (10^-11^-10^-4 ^M) of noradrenaline. Two hours later, the glycerol concentration was determined with a luminometer method and was used as an index of lipolysis. The non-selective adrenergic agonist noradrenaline represents the natural catecholamine effect and causes a concentration dependent stimulation of glycerol release which reached a plateau at the highest concentrations. The lipolytic capacity was calculated by dividing the noradrenaline-induced glycerol release per basal glycerol release. An index for the sensitivity to noradrenaline is the pD_2 _value, the negative logarithm of the EC50 value, which is the concentration of the agonist giving half of its maximum effect. This value was determined by linear regression analysis after log-logit transformation of the ascending part of the concentration-response curve. Maximal lipolytic rate of noradrenaline was used as indications of responsiveness. The lipolytic rates were also corrected for the difference in basal lipolysis between adipose tissues by subtracting the basal values. Lipolysis was expressed per number of cells incubated. Due to limited weight of biopsies in lean subjects, lipolysis experiment was only performed in biopsies from obese subjects.

### Perilipin and HSL mRNA expression

Total RNA was extracted using the RNeasy total RNA kit (Qiagen, Courtaboeuf, France). RNA concentration and purity were determined by measuring optical density at 260 and 280.nm and integrity was verified by agarose gel electrophoresis. HSL mRNA levels were measured by RT-competitive PCR as described previously [24,25]. Perilipin mRNA levels were measured by RT-PCR using β-actin as a standard. The primers sequences 5'-CTGAGCAGCCTGGCCCAGT-3' and 5'-ATTCGCTCTCGGGCTCCATC-3' were respectively used as the forward and the reverse primers for the β-actin gene and 5'-GACGAGGCCCAGAGCAAGAGA-3' and 5'-GGGTGTTGAAGGTCTCAAACA-3' for the perilipin gene.

### Immunoblot analysis

Frozen adipose tissue samples (300 mg) were homogenized with an ultra Turrax apparatus (IKA Werke, Sweden) in 0.6 ml of lysis buffer (0.25 M sucrose, 1 mM EDTA, 50 mM NaF, 1 mM benzamidine in 50 mM Tris-HCl buffer pH 7.5), with protease inhibitors (20 μg/ml leupeptin, 20 μg/ml antipain, 1 μg/ml pepstatin) and phosphatase inhibitors (okadaïc acid, phosphatase inhibitors cocktails I and II, from Sigma, L'Isle d'Abeau, France). Infranatant I (fat-free homogenate) and fat cake (FC) were separated by centrifugation (12,000 g for 20 minutes at 4°C). The fat cake was washed once more with 0.6 ml of lysis buffer (I2). Proteins present in the FC fraction were extracted twice over by centrifugation in 0.6 ml of 125 mM Tris buffer with 5% SDS, 20% glycerol, proteases and phosphatase inhibitors and both fractions were mixed together. Preliminary experiments showed that additional extractions of the fat cake proteins and the infranatant yielded no more proteins (Data not shown). Total protein contents were measured by BCA assay (Pierce, Rockford, IL). Aliquots corresponding to 40 μg and 10 μg of total proteins for I and FC fractions respectively were analyzed by SDS-PAGE and transferred to PVDF membranes along with a standard of recombinant human HSL (C. Holm, Lund, Sweden) used for normalization of the results between different immunoblots. I and FC extracts of SC and OM biopsies of a given subject were all analyzed from the same immunoblot.

Immunoblotting was performed with either an affinity-purified polyclonal rabbit anti-human HSL antibody (1:1,000) (C. Holm, Lund, Sweden) or an anti-perilipin antibody (Progen, Heidelberg, Germany 1:2,000) and the suitable secondary antibodies, before a brief incubation with enhanced chemiluminescence detection reagents (ECL, Amersham, Buckinghamshire, UK). Autoradiographs were quantified by densitometry (Imagemaster TotalLab, Amersham, Buckhinghamshire, UK) and expressed according to adipose tissue weight.

### HSL enzymatic activity

It was measured on I fractions using the diolein analogue 1(3)-mono [^3^H] oleoyl-2-oleylglycerol as substrate as described previously [24,25]. One unit of enzyme activity is defined as 1 μ mole of oleic acid released per minute at 37°C. Since the phosphorylated and dephosphorylated forms of the enzyme have the same activity toward diglyceride substrates, the total amount of activable enzyme in the sample is measured [24,25]. Samples were analyzed in triplicate and lipase activity was related to the weight of homogenized adipose tissue. Due to limited amount of tissue sampled from most lean subjects, HSL activity was not measured in these subjects.

### Statistical analysis

Results are shown as mean and SEM. Statistical analysis was performed using two-tailed Student's t-test for paired values (SC versus OM) and Mann-Whitney test (obese versus controls). P < 0.05 was considered significant. Analysis of trends was performed using linear regression.

## Results

### Clinical Characteristics of the two groups of subjects

(Table 1). As expected for obese women, plasma triacylglycerols and non-esterified fatty acids were higher than in the lean women (P < 0.05). Although the obese subjects had higher plasma insulin and glucose concentrations than controls, none had overt type 2 diabetes. Adipocyte volume was highly different between our groups of tissues: adipocytes from obese women are twice and 5-fold as big as adipocytes from lean women in SC and OM depot respectively (P < 0.01). In the lean women, the SC adipocytes volume was 420% bigger than the OM adipocytes (P < 0.05). In the obese women, the volume difference reached 210% (P < 0.05).

**Table 1:** Characteristics of lean and obese women

	Lean subjects n = 6	Obese subjects n = 10
Age, years	31.5 ± 5.7	32.1 ± 2.9

BMI, kg/m^2^	22.6 ± 1.0 (22,5-25,3)	42.0 ± 1.4** (35,4-50,2)

Pl. Triacylglycerols, mM	1.1 ± 0.2	1.9 ± 0.3*

Pl. NEFA, mM	0.60 ± 0.20	0.99 ± 0.11**

Pl. Cholesterol, mM	4.6 ± 0.3	5.5 ± 0.3

Pl. Glucose, mM	4.4 ± 0.4	5.4 ± 0.1*

Pl. Insulin mU/L	7.0 ± 1.0	16.6 ± 2.9*

Pl. Glucagon, ng/L	ND	132 ± 12

SC fat cell volume, pL	590 ± 110	1410 ± 130**

OM Fat cell volume, pL	140 ± 50 $	670 ± 120** $

Values are given as the mean ± SEM. * p < 0.05, ** p < 0.01 versus lean subjects.

$ p < 0.05 versus the corresponding value of subcutaneous tissue in the same group of subjects.

ND; not defined Pl.; plasma BMI; body mass index SC; subcutaneous adipose tissue OM; omental adipose tissue NEFA; non esterified fatty acids pL; picolitre

### HSL and *PLIN *mRNA levels

In the obese subjects, HSL mRNA level was higher in SC than in OM adipose tissue (21.5 ± 4.9 versus. 14.7 ± 2.8 attomoles/μg RNA respectively, P < 0.05). The corresponding values in the lean women were 6.8 ± 0.3 in SC adipocytes and 4.5 ± 0.3 attomoles/μg RNA in OM adipocytes (p < 0.05). In both depots, HSL mRNA abundance in the obese women were 300% higher than in the lean women (p < 0.05). In the obese subjects, *PLIN *mRNA abundance expressed relative to β-actin was significantly higher in SC than in OM adipose tissue (1.48 ± 0.24 versus. 0.75 ± 0.22, P < 0.01). The corresponding values in the lean subjects were 0.81 ± 0.07 and 0.87 ± 0.25 (P > 0.05). Therefore, values were comparable in OM adipose tissue for the obese and the lean women, but perilipin mRNA values in SC adipose tissue were significantly higher in the obese than in the lean women (P < 0.05).

### Intra-cellular content and distribution of HSL and perilipin

The Western blot analysis was performed on frozen adipose tissue. We investigated whether the freezing/thawing cycle modified the distribution of HSL and perilipin between fat-free homogenate and fat cake. Additional immunoblots were performed with freshly collected and frozen samples of the same tissue from two lean and two obese (Zucker) rats and from one obese human. Identical results were obtained (data not shown) and suggested that freezing/thawing cycle have not damaged the lipid droplets.

**Figure 1:**
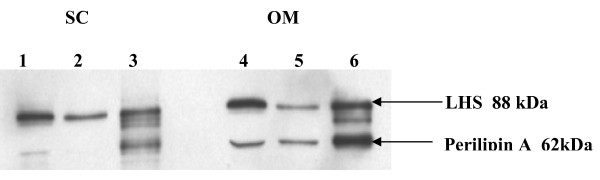
**Representative Western blot analysis of HSL and perilipin in homogenates from subcutaneous (SC) (lines 1-2-3) and omental (OM) adipose tissues (lanes 4-5-6) from an obese woman**. Lanes 1 and 4 contained perilipin and HSL proteins of fat-free infranatants (I1) (40 μg of total proteins). Lanes 2 and 5 contained proteins from the second fat-free infranatants (I2) (40 μg of total proteins). Lanes 3 and 6 contained perilipin and HSL proteins extracted from the fat cake (FC) (for more details, see research methods and procedures).

**Figure 2:**
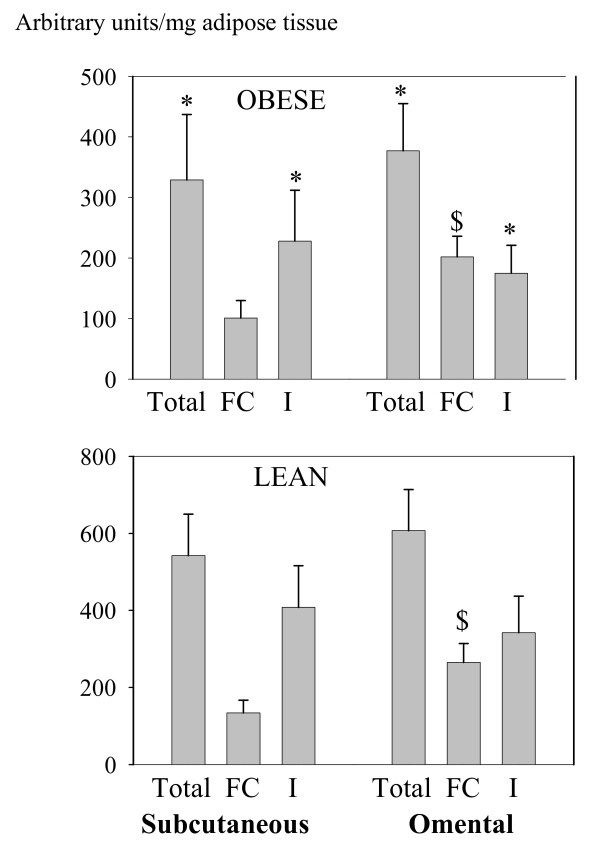
**Quantification of HSL proteins in subcutaneous and omental adipose tissues of obese (upper graph) and lean women (lower graph)**. The relative amount was measured in pooled fat-free infranatants (I: I1+I2), fat cakes (FC) and whole material (Total).* P < 0.05 versus the corresponding value in lean women, $ p < 0.05 versus the corresponding value of subcutaneous tissue in the same group of subjects.

**Figure 3:**
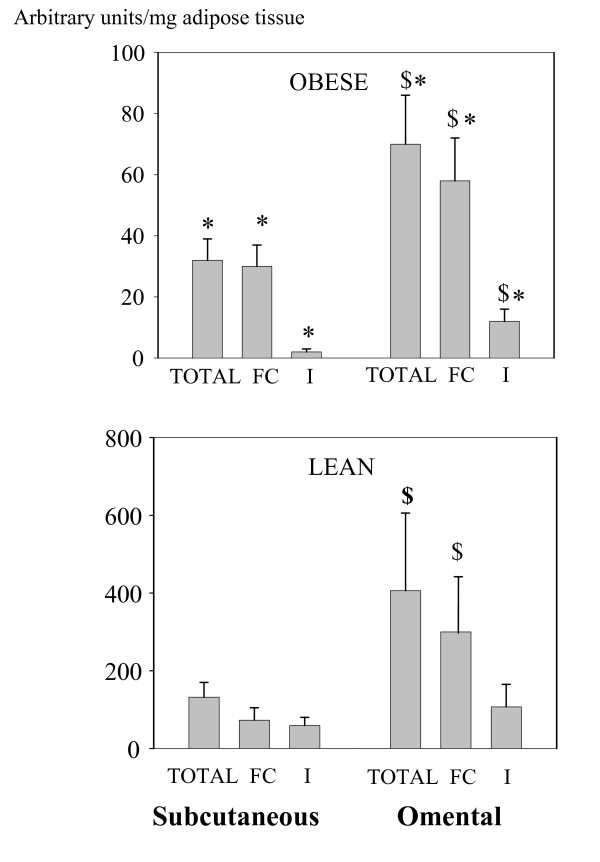
**Quantification of perilipin proteins in subcutaneous and omental adipose tissues of obese (upper graph) and lean women (lower graph)**. The relative amount was measured in pooled fat-free infranatants (I: I1+I2), fat cakes (FC) and from whole material (Total).* P < 0.05 versus the corresponding value in lean women, $ P < 0.05 versus the corresponding value of subcutaneous tissue in the same group of subjects.

Figure 1 shows a representative immunoblot of adipose tissue from an obese subject. HSL and perilipin A were detected as bands at 80 kDa and 62 kDa respectively. A band at 45 kDa was occasionally detected in FC extracts and probably represented perilipin B. HSL and perilipin A protein contents (Figures 2 and 3 respectively) were estimated in whole tissue (called "total" fractions), in fat cake fractions (FC), and in fat-free homogenates (I). The values were normalized to the weight of adipose tissue used for homogenization. In both depots, HSL and perilipin amounts were lower in the obese than in the lean subjects (P < 0.05), this difference being particularly important for perilipin. Amounts of total HSL protein were comparable between fat depots for both lean and obese subjects but the content in FC fractions of OM tissues was twice as much as that in FC fractions of SC tissues (P < 0.05) (Figure 2). Concerning perilipin A (Figure 3), all protein fractions of both depots were lower in the obese than in the lean women (from a 4 to a 10 fold reduction) (P < 0.05) Whatever the BMI, the amounts of perilipin A in OM tissue increased by 100 to 200% compared with SC adipose tissue (P < 0.05) in all fractions (total, FC and I) except in I fraction of lean subjects where the trend for higher values was not significant (P = 0.06).

**Figure 4:**
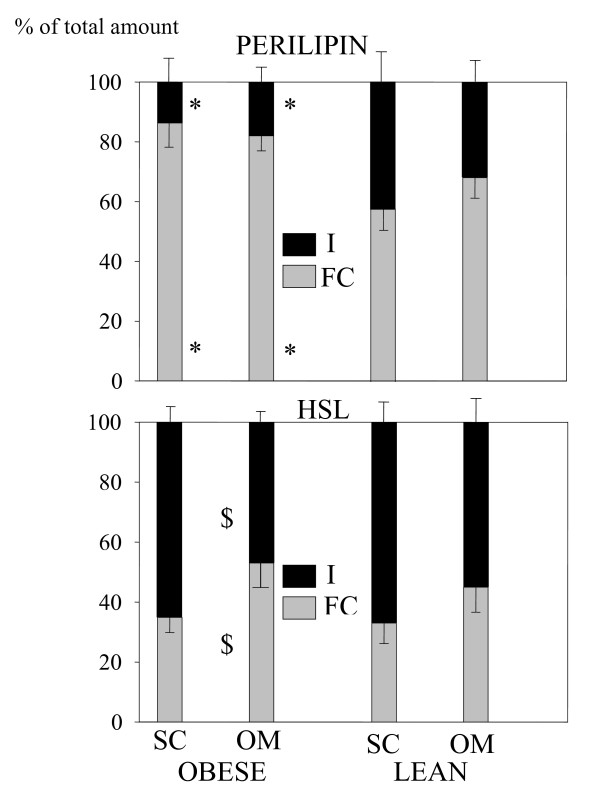
**Distribution of HSL (lower panel) and perilipin (upper panel) between fat-free homogenate (I) and fat cake (FC) in subcutaneous (SC) and omental (OM) adipose tissues of lean and obese women**. * P < 0.05 versus the corresponding value in lean subjects. $ P < 0.05 versus the corresponding value of subcutaneous tissue in the same group of subjects.

Figure 4 shows the percent cellular distribution of HSL and perilipin between fat-free homogenates and fat cake extracts. Perilipin was mostly (more than 80%) present in the fat-cake fraction in both adipose tissues of the obese subjects, this predominance in fat cake extracts being less marked (around 60%) in both depots of the lean women (P < 0.05 versus the obese). The majority (55 to 65%) of HSL protein was found in the non-lipid fractions. The HSL distribution was comparable in the lean and the obese subjects whatever the fat depot, except in lean and obese FC fractions which displayed an HSL content in OM depot twice as high as in SC fat tissue.

### HSL activity

Maximal HSL activity was measured in the fat-free homogenates of SC and OM adipose tissues in obese subjects. They were comparable (28.5 ± 0.7 vs. 27.2 ± 1.0 mU/μg of adipose tissue respectively).

### Basal and noradrenaline-stimulated lipolysis in isolated adipocytes from obese women

**Figure 5:**
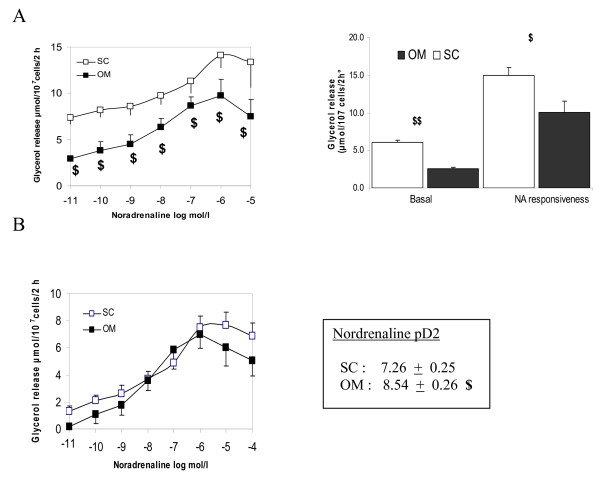
**Regional differences in basal, noradrenaline-stimulated lipolysis in adipocytes isolated from omental (OM) and subcutaneous (SC) adipose tissue**. Results are expressed as a function of cell number (A) and cell number minus basal lipolysis (B) in obese women. Data are mean ± SEM. The lipolytic capacity was calculated by dividing the noradrenaline-induced glycerol release per basal glycerol release. An index for the sensitivity to noradrenaline is the pD_2 _value, the negative logarithm of the EC50 value, which is the concentration of the agonist giving half of its maximum effect. Maximal lipolytic rate of noradrenaline was used as indications of responsiveness. It was calculated by dividing the noradrenaline-induced glycerol release by basal glycerol release. $ P < 0.05 versus the corresponding value of subcutaneous tissue in the same group of subjects $$ P < 0.01 versus the corresponding value of subcutaneous tissue in the same group of subjects.

are reported in Figure 5A, and values are expressed per cell number. Basal lipolysis was 50% lower in OM adipocytes compared with SC cells (P < 0.01). The maximal and sub-maximal lipolytic responses to noradrenaline were also lower in OM adipocytes (P < 0.05). In Figure 5B, the lipolytic rates were corrected for the difference in basal lipolysis between SC and OM fat tissue, by subtracting the basal values. The only remaining difference between both tissues was a higher sensitivity to noradrenaline of OM adipocytes, as evaluated by the pD2 value (P < 0.05).

### Correlation between perilipin protein, adipocyte volume (Figure 6A) and basal lipolysis (Figure 6B)

**Figure 6:**
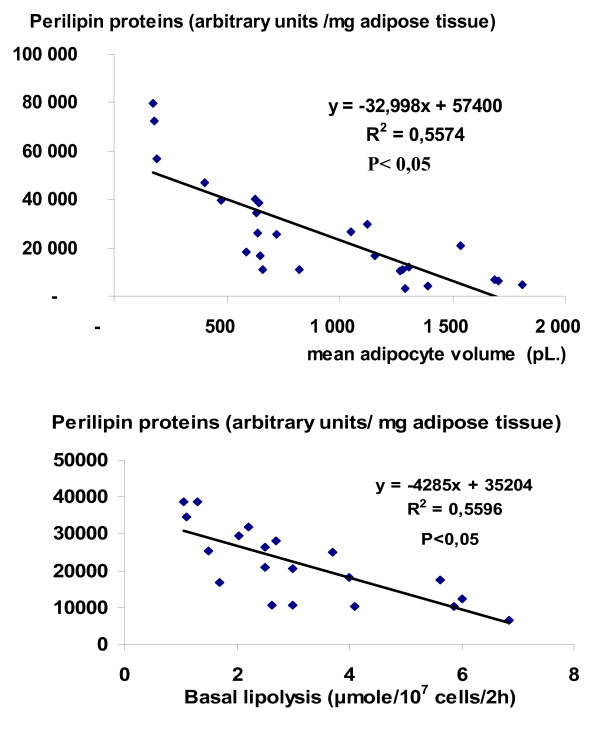
**Relationship between perilipin proteins in the whole material (SC and OM adipose tissues of lean and obese women) and adipocyte volume (upper graph) and basal lipolysis (lower graph)**. The correlation coefficient (R^2^) was calculated using linear regression analysis. (pL; picolitre)

Total perilipin protein content was negatively correlated to the volume of adipocyte isolated from both depots from the lean and the obese subjects (R^2 ^= 0.56, P < 0.05). It was also negatively correlated with basal lipolysis in both depots from the obese women (R^2 ^= 0.56, P < 0.05). A similar negative linear correlation was obtained between the mean fat cells diameter and the perilipin content in FC fractions (R^2^= 0.60, P < 0.05) (data not shown).

## Discussion

In the present study, regional differences of perilipin A and HSL expressions were examined in lean and obese women. The main results were summarized in Table 2.

**Table 2:** Summary of the results obtained in this study between lean and obese subjects and between omental and subcutaneous adipose tissues

	Perilipin		HSL		
	
Ad. tissue	mRNA	Protein	Enzymatic activity	mRNA	Protein
**SC**	Obese > lean *	Obese < lean *	ND	Obese > lean *	Obese < lean *

**OM**	Obese = lean	Obese < lean *	ND	Obese > lean*	Obese < lean *

**Obese**	SC > OM *	SC < OM *	SC = OM	SC > OM *	SC = OM

**Lean**	SC = OM	SC < OM *	ND	SC > OM *	SC = OM

* p < 0.05 ad; adipose ND; not determined Prot; proteins OM; omental SC; subcutaneous

### Depot-specific differences in HSL expression

Despite higher HSL mRNA levels in SC versus OM adipose tissues, we did not find any regional difference in HSL protein content in both lean and obese women and in HSL activity in the obese women (lean women were not analyzed for HSL activity). Some differences in HSL gene expression between fat depots have been previously reported [21,26,27]. The direction of fat-depot differences is unclear. HSL mRNA level is either identical in both depots [26,27] or higher in SC depot than in OM fat [21], like it was in the present analysis. Our data suggest that there was no depot-specific difference in HSL protein expression in our cohort. The absence of correlation between protein expression and mRNA level suggests that HSL may be regulated at some post-transcriptional levels (such as phosphorylation which activates HSL activity and induces HSL translocation from the cytosol to the lipid droplet upon adipocyte stimulation [3]).

HSL distribution between FC and non-lipid fractions in adipocytes showed a higher percentage of HSL coated the lipid droplet in OM tissue compared to SC fat from the obese women (Figure 4). We can therefore speculate that under catecholamine stimulation, 75% of HSL (the I fraction in SC adipocytes from the obese women) could move to the lipid droplet compared to 50% only in OM cells) and could be responsible for a higher hydrolysis of TAG in SC adipocytes versus OM cells. In the lean women, a trend for a similar mechanism did not reach significance. Depot difference in the HSL distribution could therefore influence HSL regulation and modulate TAG accumulation.

We estimated that 55 to 65% of the HSL proteins were localized in the fat-free homogenates in both depots in the resting state (figure 4). This is in agreement with the 60% of HSL being associated with lipids under non-stimulated conditions in cultured adipocytes [28]. As reviewed by M. Lafontant [3], the recent model suggesting that HSL is diffusively distributed throughout the cytosol in unstimulated cells and moves from the cytosol to the surface of lipid droplets where it induces lipolysis, is imperfect. According to our data, around 60% of HSL proteins were localized in the non-lipid fraction in the resting state.

### Depot-specific differences in perilipin expression and lipolysis

The present study reported regional differences in lipolysis regulation. Basal lipolysis and maximal noradrenaline-stimulated lipolysis expressed per cell number increased in SC versus OM adipocytes in the obese women. Our findings concerning a decreased basal lipolysis in OM versus SC fat depot are consistent with several previous studies [16,17,20,21,29]. As regards to pD2, an increased sensibility to noradrenaline in OM fat depot versus SC adipose tissue suggests that part of the regional differences in noradrenaline-induced lipolysis was due to site variations in β1-,2-,3- adrenoceptor subtype and/or the α-2-adrenoceptor subtype. According to noradrenaline responsiveness, SC adipocytes which displayed some higher volumes than OM adipocytes, were more lipolytic on an absolute basis. It is consistent with the notion that larger adipocytes accumulate more triglycerides and release more fatty acids than smaller adipocytes [16,30]. As SC adipocytes are generally larger than those in OM fat depot [16,17,21,31,32], it suggests that SC adipocytes displayed a higher fat storage capacity than OM adipocytes.

A 2-fold increase of perilipin protein level was found in OM versus SC adipose tissue in both obese and lean women. Conversely, perilipin mRNA levels were either increased in SC versus OM fat depot in the obese women or not statistically different in the lean women. These results suggest that perilipin may be regulated at post-transcriptional levels which could include effects on mRNA stability, transcription efficiency and phosphorylation. They confirm previous studies [12,33]. Mechanisms controlling the protein stability of perilipin are largely unknown in adipocytes. Kovsan et al. proposed that proteolysis of perilipin involving the lysosomial protein degradation machinery may constitute a novel mechanism for regulating adipocyte lipolysis [34]. Perilipin subcellular distribution did not show any differences between fat depots whatever the BMI (figure 4).

Up to date, depot-specific differences in perilipin expression were only investigated in two studies involving human [12,33]. Both studies do not find any significant difference of perilipin content between both fat depots in obese subjects. The discrepancy between these studies and our results could reside in gender differences since the material in previous studies is composed of adipose tissues from males and females [12,33]. Actually Wang et al [12] found higher perilipin protein content in SC adipocytes from obese men compared with women. We think therefore that it was more relevant to study perilipin expression either in men or in women (as it is in the present study).

The increased perilipin protein expression in OM fat depot versus SC adipose tissue was negatively correlated to the mean adipocyte volume (in the lean and the obese women) and to basal lipolysis in the obese women (Figure 5). This point will be focused below.

### Perilipin and HSL expressions in obese women

In the present study, obesity was associated with a decreased perilipin and HSL proteins in both adipose tissues. HSL mRNA levels were elevated in both fat depots whereas perilipin gene expression was either similar or increased in SC and OM depots respectively. The discrepancy between mRNA and proteins suggests once again that post-transcriptional regulation of perilipin and HSL occurred in adipocytes. Due to limited weight of biopsies, lipolysis experiments were not performed in the lean women. Therefore the relationship between adipocyte lipolysis and perilipin expression were not studied. However, it is commonly accepted that the action of catecholamines is impaired in obesity and that basal rate of lipolysis is increased in enlarged fat cells of obese subjects whatever the fat depot [3,4,21]. If perilipin A functions to restrain basal lipolysis [9], we hypothesized that a decreased perilipin A protein content contributes to elevated basal lipolysis in large adipocytes of obese women.

Perilipin A expression was only measured in a few studies involving obese humans [11-13,31]. Most of them found that the perilipin protein content decreases in SC adipose tissue of obese patients [11,12] and is associated with a reduced perilipin mRNA level [12,31]. Only one reported an increased mRNA and protein contents [13]. The present data strengthen the findings towards a reduced perilipin A protein expression in human obesity.

Perilipin proteins were mostly present on the lipid droplet (80% in obese women and it decreased to 60% in controls). It is consistent with the hypothesis that in the resting state (non-activated conditions), perilipins coat the lipid droplet and prevent lipases from TAGs hydrolysis, an action commonly described as "the barrier function between lipases and stored neutral lipid" [3]. An enhanced percentage of perilipin protein was found on the lipid droplet of obese women whatever the fat depot. Our results underlined differences in both the perilipin A protein expression and its subcellular distribution in obesity. Up to date no other study investigated perilipin intracellular distribution in the obese versus the lean subjects. The difference in intra-cellular perilipin A distribution between lean and obese subjects could be linked to the higher volume of adipocytes in obese versus lean (and in SC versus OM fat depot) and hence of lipid droplets, in obese subjects and (in SC fat depot), offering more surfaces for perilipin to anchor. A negative linear correlation was found between perilipin protein content (Total and FC fractions) and either the mean adipocyte volume in all biopsies or basal lipolysis in obese women. These results suggest that the total content of perilipin, the perilipin content in FC fractions, and the adipocyte size are strongly interrelated in SC as well as in OM tissues.

## Conclusions

A reduced perilipin protein expression may contribute to the higher basal lipolytic rate and may contribute to adipocyte enlargement. Regional differences in perilipin expression between OM and SC adipose tissue metabolism were found in women over the range of body mass index. Perilipin protein expression may determine lipolytic differences between SC and OM fat depots and may regulate lipid accumulation in adipocytes. Regional difference influence HSL distribution in the obese women.

## Competing interests

The authors declare that they have no competing interests.

## Authors' contributions

Conceived and designed the experiment: HR, VF, MB, VL. Performed the experiments: HR, CP. Analyzed the data: HR, MB, VL. Wrote the paper: HR, MB, VL. All authors read and approved the final manuscript.
